# Modification of the Internal Suture Technique for Mallet Finger

**DOI:** 10.1097/MD.0000000000000536

**Published:** 2015-02-13

**Authors:** Bo Jiang, Peiji Wang, Yong Zhang, Jiaju Zhao, Qirong Dong

**Affiliations:** From the Department of Hand and Foot Surgery, The Second Affiliated Hospital of Soochow University, Suzhou, Jiangsu, PR China.

## Abstract

This article describes a treatment of tendinous mallet finger deformities using a modified internal suture technique for the stable fixation of the terminal extensor tendon and bone.

Between March 2011 and July 2013, 15 patients with mallet fingers who had been treated using this modification were included in this study. The patients included 10 men and 5 women with a mean age of 33 years (range, 19–50 years). Of these patients, 9 had chronic mallet fingers, 3 were unable to comply with a splinting regimen, and 3 had a history of unsuccessful splinting therapy. The mean time between the injury and surgery was 5.5 months (range, 1–15 months). We graded the results using Crawford criteria.

The mean follow-up period was 12 months (range, 9–16 months). The mean final active range of motion of the distal interphalangeal joint flexion was 73° (range, 60°–90°). Based on Crawford evaluation criteria, 8 patients were graded as excellent, 6 were graded as good, and 1 was graded as fair. Apart from 2 documented mild nail deformities, no complications were encountered.

This modified technique should be considered for the management of a tendinous mallet finger deformity when the internal suture technique is planned.

## INTRODUCTION

Mallet fingers are common injuries and involve disruption of the terminal extensor mechanism overlying the DIPJ. Usually, mallet finger injuries occur in the work environment or during sports. Although the patient can passively extend the distal phalanx, active extension is not possible, which can result in functional and aesthetic problems. In addition, the resulting imbalance can lead to an early or late swan-neck deformity. Therefore, it is essential to restore the integrity of the terminal extensor mechanism in the DIPJ.

Mallet fingers include 2 types, tendinous and bony, with tendinous injuries being more difficult to treat.^1^ Although conservative treatment for an acute mallet finger of tendinous origin is effective in many cases, a single splint only cures or significantly improves approximately 50% of cases.^1^ The treatment failure results in chronic mallet finger and surgery is recommended.^2–5^ Surgical treatment is indicated to correct chronic mallet finger deformity for pain, dysfunction, or aesthetics. In addition, a surgical approach is also indicated for patients who are unable to comply with a splinting regimen or who have a history of unsuccessful splinting therapy.^6^ To our knowledge, there are no clearly established criteria for an acceptable result.

Bauze and Bain^7^ reported an internal suture technique that allows for the accurate realignment of the tendon–bone interface. However, the technique is likely to include the dermis or neurovascular structure and cause suture loosening. Based on these considerations, we present a modification of the internal suture technique in this study. The results and advantages of the procedural modifications are reported.

## MATERIALS AND METHODS

This study was approved by our Institutional Review Board, and all patients were available for review. This study was performed from March 2011 to July 2013. Fifteen patients with a mallet finger of tendinous origin were treated using a modification of the internal suture technique with transarticular K-wire fixation. Patients with no limitation in passive motion of the DIPJ and proximal interphalangeal joints and no swan-neck deformity were enrolled in this study. The patients were assessed for functional recovery and incidence of complications. The ROM of the DIPJ of finger involved was recorded using a forearm goniometer. The functional outcomes were assessed using Crawford criteria (Table 1).^8^

**TABLE 1:**
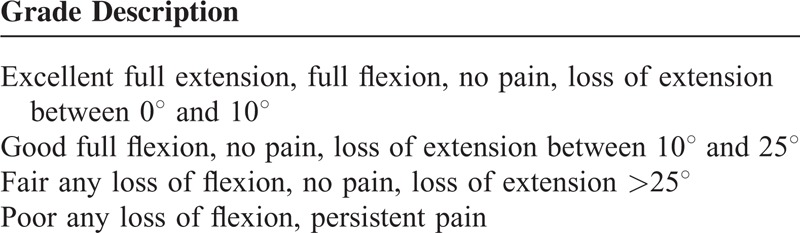
Outcome Assessment: Crawford Evaluation Criteria

Preoperative radiographs were obtained in all cases. Patients were excluded if the injury was a bony mallet finger injury. The inclusion criteria for this study were chronic mallet fingers (>3 months delay from the injury without treatment), inability to comply with a splinting regimen, and history of unsuccessful splinting therapy.

### Surgical Technique

**FIGURE 1:**
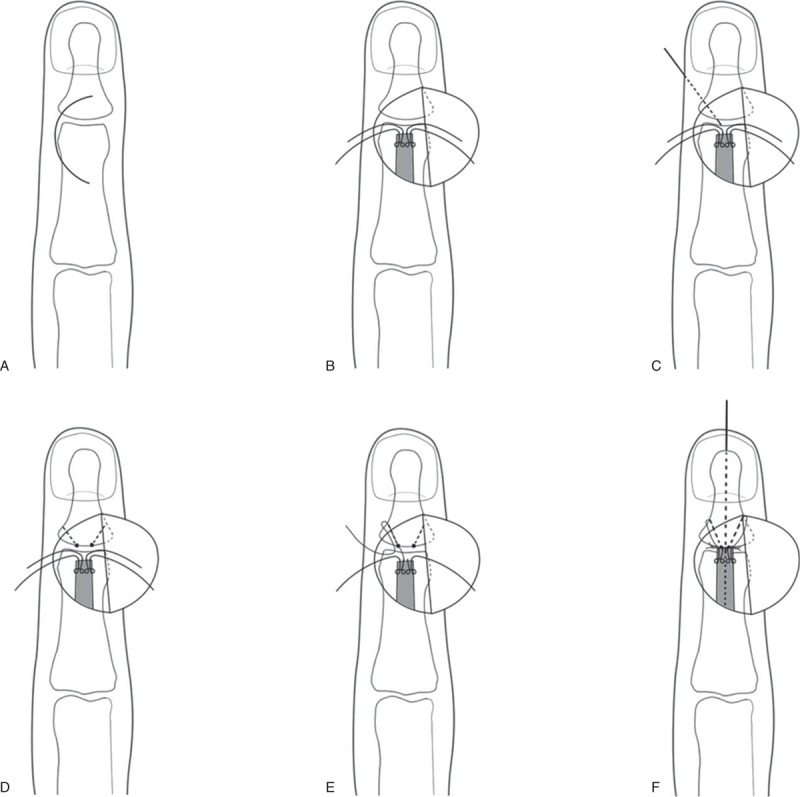
Diagram showing the surgical technique of the modified internal suture.

**FIGURE 2:**
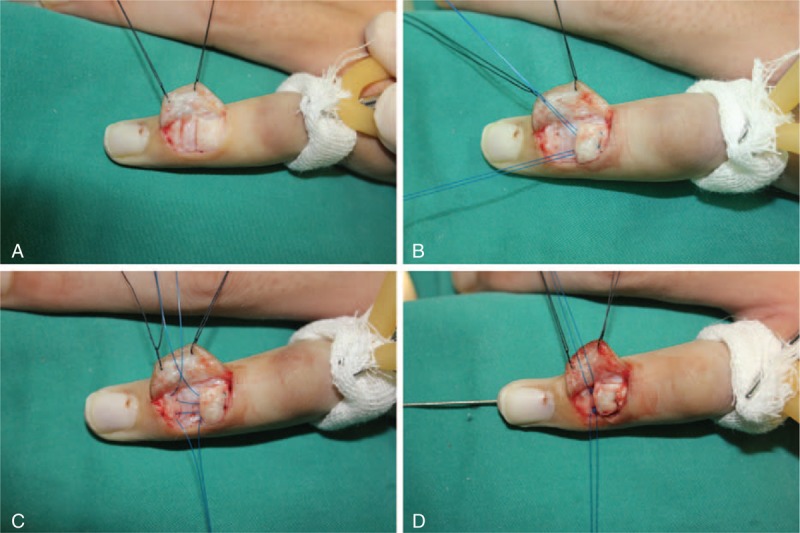
Case 1. A. The scar tissue between the terminal extensor tendon and distal phalanx insertion was excised. B. Two Kessler sutures with 4–0 Prolene were passed from the extensor tendon, and 2 bone holes were drilled. C. The suture was passed through the drill hole and reentered through the dorsal wound along the periosteal surface. D. After inserting a K-wire to fix the DIPJ, knots were tied over the dorsal aspect of DIPJ. DIPJ = distal interphalangeal joint.

The procedure was performed under brachial plexus block or digital block. A transverse or longitudinal C-shaped incision was made over the dorsal aspect of the DIPJ (Figure 1A). A flap was raised and the terminal extensor mechanism was exposed. After the excision of the scar tissue between the terminal extensor tendon and distal phalanx insertion, the dorsal cortex was peeled off at the base of the distal phalanx. Two Kessler sutures with 4–0 Prolene were passed from the extensor tendon in a standard manner, and the needles were straightened (Figure 1B). Then, 2 drill holes on the base of the distal phalanx were created bicortex obliquely in a dorsal to lateral median line direction by a 0.8-mm K-wire (Figures 1C and D). One straight needle with the Prolene suture was driven along the ipsilateral drill hole on the distal phalanx and exited from the lateral median line. A small stab incision of 2 to 3 mm was made over the needle down to the periosteum of the distal phalanx. The needle was pulled out at the stab incision. The stab incision allowed the needle to be passed back through the same track without catching the dermis or neurovascular structure. The goal was to reenter the dorsal wound via a different path (going along the periosteum) to catch the bone for fixation (Figure 1E). The second suture was placed on the other side of the distal phalanx using the same technique. Finally, a 1.0-mm K-wire was advanced longitudinally from the tip of the finger into the middle phalanx to hold the DIPJ in slight extension. The extensor tendon was advanced onto the drill holes of the distal phalanx by placing traction on the extensor tendon using a skin hook. Both Prolene sutures are then tied (Figure 1F). Before tying the knots, it was confirmed that the Prolene sutures were lying securely on the tendon insertion point of the distal phalanx. The dorsal incision was closed with 5–0 Prolene stitches, and the lateral stab incisions did not require closure (Figure 2).

## RESULTS

All procedures were conducted by a single surgeon. This study was composed of 10 men and 5 women with a mean age of 33 years (range, 19–50 years). The little finger was the most commonly affected (6 cases), followed by the ring finger (4 cases), long finger (3 cases), and index finger (2 cases). Of these patients, 9 were chronic mallet fingers (>3 months delay from the injury without treatment), 3 were unable to comply with a splinting regimen, and 3 had a history of unsuccessful splinting therapy. The mean delay between surgery and injury was 5.5 months (range, 1–15 months). Data for each patient are included in Table 2.

**TABLE 2:**
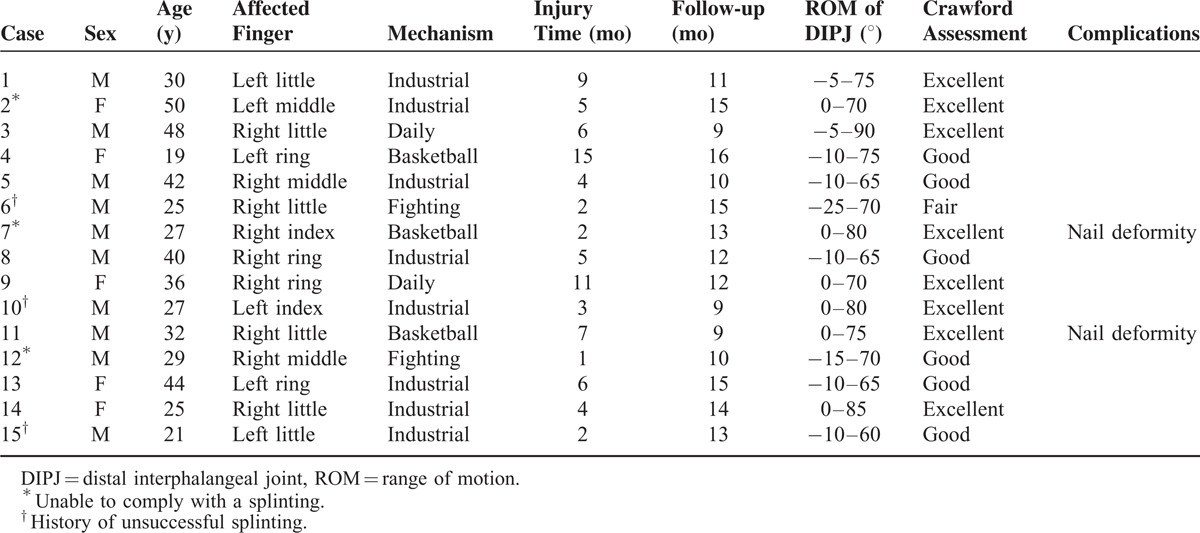
Patient Data and Results

**FIGURE 3:**
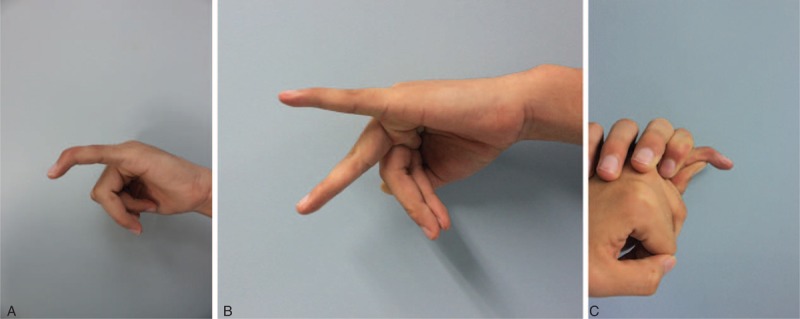
Case 1. A. Preoperative view of the patient with chronic tendinous mallet finger deformity. B and C. Photographic view taken at 11 months postoperatively, showing the range of DIP joint motion. DIP = distal interphalangeal.

**FIGURE 4:**
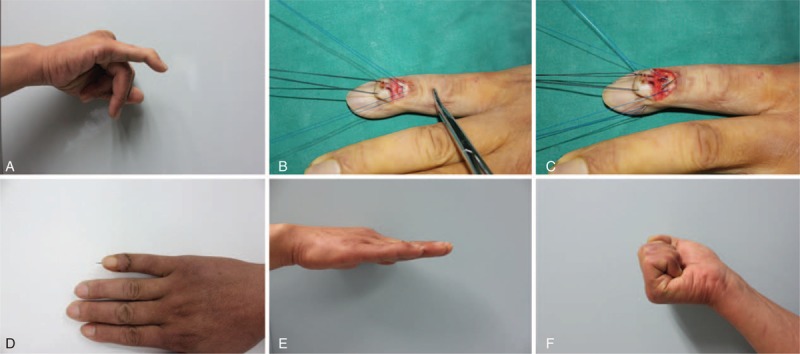
Case 3. A. Preoperative view of the patient with chronic mallet finger deformity of tendinous origin. B. A transverse C-shaped incision was made and a flap was raised. Two Kessler sutures with 4–0 Prolene were passed from the extensor tendon, and the needles were straightened. C. The 2 straight needles were passed back the dorsal wound by both sides of small stab incisions. D. Postoperative view at 3 weeks. E and F. Photographic view taken at the final follow-up, which shows the ROM of DIPJ. DIPJ = distal interphalangeal joint, ROM = range of motion.

The follow-up period ranged from 9 to 16 months (average, 12 months). At 6 weeks postoperative, the K-wire was removed in all patients. Active and passive DIPJ exercises were initiated immediately after removal of the K-wire. Two patients developed mild nail deformities. No other complications were encountered. The mean final extensor lag was 6.6°, and the average mean final active range of motion of the DIPJ flexion was 73° (range, 60°–90°) (Figures 3 and 4). None of the patients complained of subjective pain of the injury finger. According to Crawford criteria, 8 patients were graded as excellent, 6 were graded as good, and 1 was graded as fair (Table 2).

## DISCUSSION

Tendinous mallet finger deformity is usually treated with prolonged, continuous splint usage. However, without treatment or with failed nonsurgical treatment, the injury can result in chronic mallet finger, which impacts the aesthetics and function of the injured finger.^9^ In addition, in some patients with chronic mallet deformity, swan-neck deformities might occur as a result of the terminal extensor mechanism imbalance.

It is recommended to treat chronic tendinous mallet finger surgically. A surgical approach is also indicated for patients with tendinous injuries who are unable to comply with splinting or who have a history of unsuccessful splinting.^10^ The tendinous injury is a challenging condition, and treatment recommendations are limited.^6^ Methods that have been described as treatment for the tendinous injuries include scar excision and end-to-end tenorrhaphy,^2^ Fowler central slip release,^3^ shortening suture,^4^ tenodermodesis,^11^ the use of a mini bone anchor,^12^ the deepithelialized pedicled skin flap technique,^13^ central slip tenotomy,^5^ the Thompson procedure,^14^ and the pull-in suture technique.^6,15^ However, infection, skin necrosis, nail deformity, incomplete correction of the extensor lag, and limitations in flexion are major complications according to the type of surgery.^9^ Currently, there are no clearly established criteria for the treatment of chronic mallet finger.

Bauze and Bain^7^ used an internal suture technique for precise restoration of the bone–tendon integrity to treat tendinous mallet finger. The sutures were used to catch soft-tissue septae for the fixation of terminal extensor mechanisms. A satisfactory treatment outcome was achieved. However, in our experience, this technique is likely to catch the dermis or neurovascular structure, which may cause pain or altered sensibility of the injured finger because of neuromas. Complications of the internal suture technique include nail deformity, superficial infection, and pin-track infection.^7^ In addition, according to our experience, the sutures did not necessarily run along the periosteum of the base of the distal phalanx because it was likely that the suture would loosen when the soft-tissue septae were caught for fixation. To prevent these complications and problems, we modified the original internal suture technique.

The technique presented in this study used 2 Prolene sutures passed through 2 drilled oblique bone holes on the base of the distal phalanx. Because the sutures were passed back along the periosteum of the distal phalanx, the bone was caught for fixation. The surgical treatment provided a stable fixation of the extensor tendon and bone, which can eliminate suture loosening. The soft tissue such as the dermis or neurovascular structure did not get caught; therefore, there was no pain or altered sensibility at the finger. No severe complications, such as skin necrosis or pin-track infection, were observed. The patients in this study achieved a satisfactory treatment outcome (with only 1 fair result), with a mean extensor lag of 6.6° and a mean active DIPJ flexion of 73°. This modified technique was more reliable and functional than the original method.

There were 2 limitations in our study. First, drilling bone holes obliquely in a dorsal to lateral median line direction requires a delicate surgical technique and is the main disadvantage of this technique. Second, during the study period, we did not use other surgical methods. Thus, no comparison between techniques could be made.

This study was designed to document the results of a modified internal suture technique for the treatment of tendinous mallet finger deformity. Satisfactory results with only 1 fair result were obtained by using the modified technique. According to our experience, this technique provides an alternative and acceptable treatment modality for the treatment of a tendinous mallet finger.
